# Repression of mitochondrial metabolism for cytosolic pyruvate-derived chemical production in *Saccharomyces cerevisiae*

**DOI:** 10.1186/s12934-019-1226-6

**Published:** 2019-10-15

**Authors:** Keisuke Morita, Fumio Matsuda, Koji Okamoto, Jun Ishii, Akihiko Kondo, Hiroshi Shimizu

**Affiliations:** 1 Graduate School of Information Science and Technology, Osaka University, 1-5 Yamadaoka, Suita, Osaka 565-0871 Japan; 2 Graduate School of Frontier Bioscience, Osaka University, 1-3 Yamadaoka, Suita, Osaka 565-0871 Japan; 3 Engineering Biology Research Center, Kobe University, 1-1 Rokkodai, Nada, Kobe, Hyogo 657-8501 Japan; 4 Graduate School of Science, Technology and Innovation, Kobe University, 1-1 Rokkodai, Nada, Kobe, Hyogo 657-8501 Japan; 5 Department of Chemical Science and Engineering, Graduate School of Engineering, Kobe University, 1-1 Rokkodai, Nada, Kobe, Hyogo 657-8501 Japan; 6 RIKEN Center for Sustainable Resource Science, 1-7-22 Suehiro, Tsurumi, Yokohama, Kanagawa 230-0045 Japan

**Keywords:** *Saccharomyces cerevisiae*, Mitophagy, Metabolome, Mitochondrial pyruvate carrier, ^13^C-metabolic flux analysis, 2,3-Butanediol

## Abstract

**Background:**

*Saccharomyces cerevisiae* is a suitable host for the industrial production of pyruvate-derived chemicals such as ethanol and 2,3-butanediol (23BD). For the improvement of the productivity of these chemicals, it is essential to suppress the unnecessary pyruvate consumption in *S. cerevisiae* to redirect the metabolic flux toward the target chemical production. In this study, mitochondrial pyruvate transporter gene (*MPC1*) or the essential gene for mitophagy (*ATG32*) was knocked-out to repress the mitochondrial metabolism and improve the production of pyruvate-derived chemical in *S. cerevisiae*.

**Results:**

The growth rates of both aforementioned strains were 1.6-fold higher than that of the control strain. ^13^C-metabolic flux analysis revealed that both strains presented similar flux distributions and successfully decreased the tricarboxylic acid cycle fluxes by 50% compared to the control strain. Nevertheless, the intracellular metabolite pool sizes were completely different, suggesting distinct metabolic effects of gene knockouts in both strains. This difference was also observed in the test-tube culture for 23BD production. Knockout of *ATG32* revealed a 23.6-fold increase in 23BD titer (557.0 ± 20.6 mg/L) compared to the control strain (23.5 ± 12.8 mg/L), whereas the knockout of *MPC1* revealed only 14.3-fold increase (336.4 ± 113.5 mg/L). Further investigation using the anaerobic high-density fermentation test revealed that the *MPC1* knockout was more effective for ethanol production than the 23BD production.

**Conclusion:**

These results suggest that the engineering of the mitochondrial transporters and membrane dynamics were effective in controlling the mitochondrial metabolism to improve the productivities of chemicals in yeast cytosol.

## Background

Yeast *Saccharomyces cerevisiae* is one of the suitable microbial industrial hosts for bioethanol production due to its robustness to pH stress and fermentation ability [1]. Moreover, it has been applied in industrial productions of various chemicals from glucose. Pyruvate produced via glycolysis acts as a key precursor for the biosynthesis of various chemicals including ethanol, lactate, and 2,3-butanediol (23BD). 23BD is known to be an antifreeze agent and a raw material for synthetic rubber [2–5]. In order to improve the productivity of these chemicals, it is essential to suppress the unnecessary pyruvate consumption in the *S. cerevisiae* cells to redirect the metabolic flux toward the target chemical production pathways.

In *S. cerevisiae* cells, a large amount of pyruvate is consumed by the synthesis of cell components, ethanol production, and respiration in the mitochondrion. Several studies reported that the knockout or control of ethanol biosynthesis pathway genes in *S. cerevisiae* improves the production of the pyruvate-derived chemicals [3, 4, 6, 7]. In *Escherichia coli*, decreasing the citrate synthase expression by CRISPER/Cas9 system increased the *n*-butanol yield [8]. Moreover, a previous study reported that the repression of tricarboxylic acid (TCA) cycle by knockout of *LPD1* coding the component of the pyruvate dehydrogenase and 2-oxoglutarate dehydrogenase enhanced the cytosolic isobutanol production [9]; however, few reports have emphasized on the repression of mitochondrial metabolism in yeast. In mitochondrion, the carbon loss occurs in the TCA cycle associated with the respiratory chain under aerobic conditions. In recent years, it has been reported that mitochondrial metabolic pathways function even in anaerobic fermentation conditions such as brewing for organic acid production [10]. Therefore, the repression of mitochondrial metabolism may reduce carbon loss and is presumed to improve the productivity of pyruvate-derived target chemicals.

In this study, two approaches were investigated in order to repress the mitochondrial metabolism. The first approach includes a knockout of the mitochondrial pyruvate carrier (MPC) genes responsible for pyruvate transport to mitochondria from the cytosol. Yeast has three pyruvate transporter proteins (Mpc1p, Mpc2p, and Mpc3p) that form two types of complexes for different functions. MPC complex with Mpc1p and Mpc2p acts as an active pyruvate transporter in the aerobic condition, whereas the complex with Mpc1p and Mpc3p acts as a low active transporter under anaerobic conditions [11]. Park et al. [12] reported that enhancing the MPC gene expression increased the pyruvate uptake into mitochondria and improved the isobutanol titer produced by the valine biosynthesis pathway and heterologous Ehrlich pathway in mitochondria. In this study, the knockout of *MPC1* was emphasized to shut-off the entrance of mitochondrial metabolism because it was essential for the formation of either complex.

The second approach includes the inhibition of mitochondrial selective degradation, termed as “mitophagy”. Mitophagy is one of the autophagy mechanisms that degrade the damaged mitochondria by oxidative stress of reactive oxygen species (ROS). Molecular mechanisms of mitophagy have been actively studied using yeast as a model organism and have revealed that the Atg32p phosphorylation is indispensable for induction of mitophagy in *S. cerevisiae* [13, 14]. It has been suggested that membrane kinetics of mitophagy is also associated with fermentative metabolism. Shiroma et al. [15] reported that the ethanol production titer of *ATG32* knockout strain (mitophagy inhibited yeast strain) was enhanced in the ginjo-sake brewing conditions with low nutrient. They considered that the ethanol production was increased by excess carbons from the suppression of cell growth due to the lack of reused-nutrients supplied by the mitochondrial degradation. It is presumed that metabolic effect of *ATG32* knockout to increase the ethanol production can also be applied to improve other pyruvate-derived chemical productions.

**Fig. 1:**
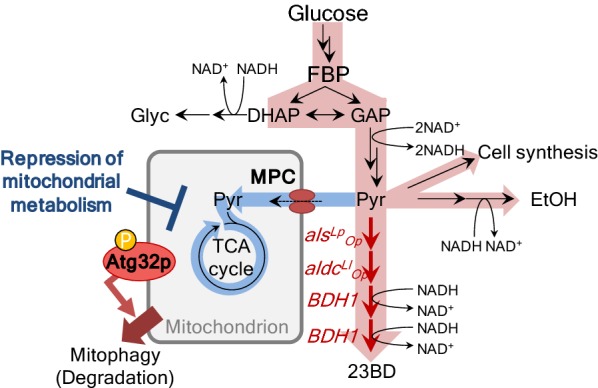
Metabolic pathways related to this research. Repression of mitochondrial pyruvate consumption is presumed to increase the growth rate and pyruvate-derived chemical production of ethanol and 2,3-butanediol

In this study, to enhance the productivity of pyruvate-derived chemicals in yeast by repressing the mitochondrial metabolism, knockout of *MPC1* and *ATG32* was carried out for the inhibition of pyruvate uptake into mitochondrion and mitophagy, respectively. The ^13^C-metabolic flux analysis (^13^C-MFA) and metabolome analysis of central carbon metabolism were performed to investigate their effect on yeast metabolism. Furthermore, in order to verify that the repression of mitochondrial metabolism was effective in improving the pyruvate-derived chemical productivity, 23BD production strains were constructed (Fig. 1). This chemical is generally preferred for yeast metabolic engineering [4, 6, 16, 17] and was used in this study due to its easy handling in metabolic engineering and low cell toxicity [18, 19].

## Result

### Fermentation profiles of *mpc1Δ* and *atg32Δ* strains in the exponential growth phase

To investigate the effect of *MPC1* and *ATG32* knockout on yeast, three strains (SCM001, SCM053, and SCM003, Table 1) were obtained by introducing a plasmid pGK426 (an empty vector with *URA3* marker) into the *S. cerevisiae* BY4742, *MPC1* and *ATG32* knockout strains, respectively. Strains were inoculated into 5 mL of SD medium containing 20 g/L of glucose in the test tube with an initial OD_600_ = 0.5 in order to obtain the fermentation profiles in a mid-log growth phase.

**Table 1:** Yeast strains and plasmids used in this study

Strain name	Description	Source or references
BY4742	*MATα*, *his3Δ1*, *leu2Δ0*, *lys2Δ0*, *ura3Δ0*	Thermo Scientific
mpc1Δ	BY4742 *mpc1Δ*::*kanMX4*	Thermo Scientific
atg32Δ	BY4742 *atg32Δ*:: *kanMX4*	Thermo Scientific
SCM001	BY4742/pGK426	This study
SCM053	mpc1Δ/pGK426	This study
SCM003	atg32Δ/pGK426	This study
SCM043	BY4742/pATP426-als^Lp^_Op_-aldc^Ll^_Op_-BDH1	This study
SCM051	mpc1Δ/pATP426-als^Lp^_Op_-aldc^Ll^_Op_-BDH1	This study
SCM042	atg32Δ/pATP426-als^Lp^_Op_-aldc^Ll^_Op_-BDH1	This study

Plasmid	Description	Source or reference
pGK426	Yeast expression vector containing *PGK1* promoter, 2*μ* origin, *URA3* marker, no expression (control plasmid)	Ishii et al. [33]
pATP426	Yeast three gene expression vector containing *ADH1*, *TDH3*, and *PGK1* promoters, 2*µ* origin, *URA3* marker, no expression.	Ishii et al. [34]
pATP426-als^Lp^_Op_-aldc^Ll^_Op_-BDH1	pATP426, co-expression of codon-optimized *L. plantarum* ALS, *L. lactis* ALDC and *S. cerevisiae BDH1* genes controlled by *ADH1*, *TDH3*, and *PGK1* promoters	Ishii et al. [6]

Table 2 shows the growth rate, glucose consumption rate, and production rate of ethanol and glycerol in the mid-log growth phase. These values were obtained from the fermentation profile (Additional file 1: Figure S1). The growth rates of both the *mpc1Δ* and *atg32Δ* strains were significantly improved (0.25 ± 0.01 h^−1^ and 0.24 ± 0.01 h^−1^, respectively) compared to that of the wild-type strain (0.15 ± 0.01 h^−1^). Although no statistically significant difference was observed between the ethanol production rate, *mpc1Δ* and *atg32Δ* strains exhibited larger value (19.9 ± 2.0 mmol gDCW^−1^ h^−1^ and 18.8 ± 1.4 mmol gDCW^−1^ h^−1^, respectively) than the wild-type strain (18.2 ± 1.2 mmol gDCW^−1^ h^−1^). In addition, the glycerol production rates of both gene knockout strains were doubled for the wild type strains. These results clearly indicate that the knockout of *MPC1* and *ATG32* altered the yeast metabolism; however, similar glucose consumption rates were observed for the wild type, *mpc1Δ*, and *atg32Δ* strains (15.7 ± 2.4 mmol gDCW^−1^ h^−1^, 14.8 ± 1.1 mmol gDCW^−1^ h^−1^, and 15.7 ± 0.9 mmol gDCW^−1^ h^−1^, respectively), suggesting that these metabolic perturbations did not occur due to the change in glucose uptake rate. These results indicated that the growth rate improvement in the *MPC1* or *ATG32* knockout strain was due to the change of the carbon flux distribution in the cell.

**Table 2:** Growth, glucose consumption, ethanol, and glycerol production rate in BY4742 wild-type, *atg32*Δ, and *mpc1Δ* strains

	SCM001 (Wild-type)	SCM053 (*mpc1Δ*)	SCM003 (*atg32Δ*)
Growth rate (h^−1^)	0.15 ± 0.01	0.25 ± 0.01	0.24 ± 0.01
Glucose consumption rate (mmol gDCW^−1^ h^−1^)	15.7 ± 2.4	14.8 ± 1.1	15.7 ± 0.9
Ethanol production rate (mmol gDCW^−1^ h^−1^)	18.2 ± 1.2	19.9 ± 2.0	18.8 ± 1.4
Glycerol production rate (mmol gDCW^−1^ h^−1^)	0.5 ± 0.3	1.3 ± 0.1	1.2 ± 0.3

### ^13^C-metabolic flux analysis of *atg32Δ* and *mpc1Δ* strains

^13^C-metabolic flux analysis (^13^C-MFA) was carried out to investigate the distributions of the intracellular carbon fluxes. *S. cerevisiae* cells were cultured using 5 mL of SD medium containing 20 g/L of [1-^13^C] glucose as a sole carbon source in a test tube. Yeast cells at the mid-log growth phase were harvested to obtain the hydrolyzed amino acids from the proteins. The ^13^C enrichment of amino acids was measured via gas chromatography-mass spectrometry. Metabolic flux distributions were estimated from the ^13^C enrichment and specific rate data (Table 2) using the yeast central metabolic model presented in Additional file 1: Table S1 (see “Methods” for detailed procedure).

**Fig. 2:**
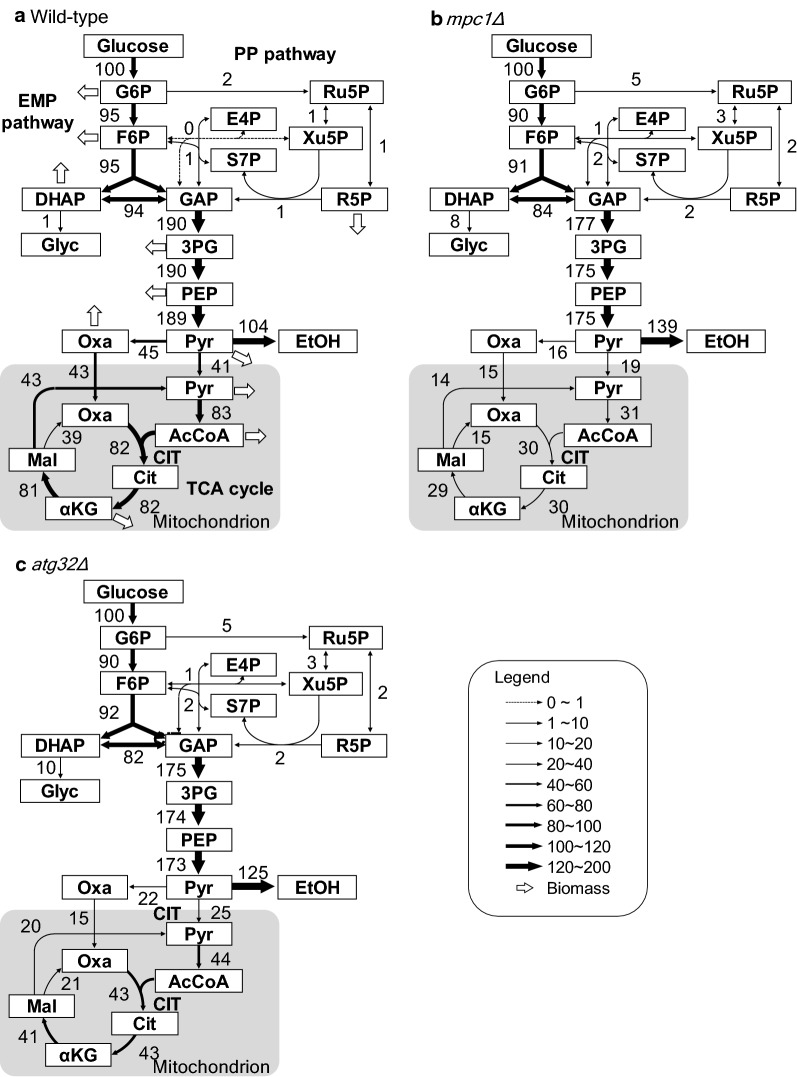
Flux distributions in BY4742 wild-type, *atg32Δ*, and *mpc1Δ* strains. Flux distributions are depicted for **a** strain SCM001 (wild-type strain), **b** strain SCM053 (*mpc1Δ* strain) and **c** SCM003 strain (*atg32Δ* strain) in the exponential growth phase. The flux values of the best fit were normalized to a specific glucose uptake rate of 100. The line width reflects the flux rate

Figure 2 depicts the metabolic flux distribution of wild-type, *mpc1Δ*, and *atg32Δ* strains in the mid-log growth phase calculated by ^13^C-MFA. The flux distributions were normalized to the glucose uptake rate (100%). Most of the glucose was metabolized by the Embden–Meyerhof–Parnas pathway (EMP pathway), and the pentose-phosphate pathway (PP pathway) flux in both *mpc1Δ* strain and *atg32Δ* strain increased from 2 to 5%. Maximum flux changes were observed in the TCA cycle. The metabolic flux from the acetyl-CoA in the TCA cycle was 82% in the wild-type strain; in contrast, it was reduced to 30% and 43% in the *mpc1Δ* and *atg32Δ* strains, respectively. To estimate the metabolic flux level of the TCA cycle, the confidence interval of the citrate synthase (CIT) flux was evaluated by the grid search method. The possible ranges of the CIT flux of the wild-type, *mpc1Δ*, and *atg32Δ* strains were 81–83%, 29–33%, and 41–45%, respectively. From these results, the TCA cycle flux of the *mpc1Δ* and *atg32Δ* strains was clearly decreased, indicating that these gene knockouts reduced the mitochondrial metabolism and redirected the saved carbon flux to the ethanol production or biomass formations.

### Intracellular metabolite analysis of *atg32*Δ and *mpc1Δ* strains

In order to investigate the effect of the *MPC1* and *ATG32* knockout on the repression of the yeast mitochondrial metabolisms, the metabolic profiles of glycolytic intermediates after fermentation were analyzed. Cells in the mid-log growth phase were inoculated to the fresh medium with initial OD_600_ = 1 and harvested after 24 h for the LC–MS/MS analysis of extracted glycolytic intracellular metabolite pools.

**Fig. 3:**
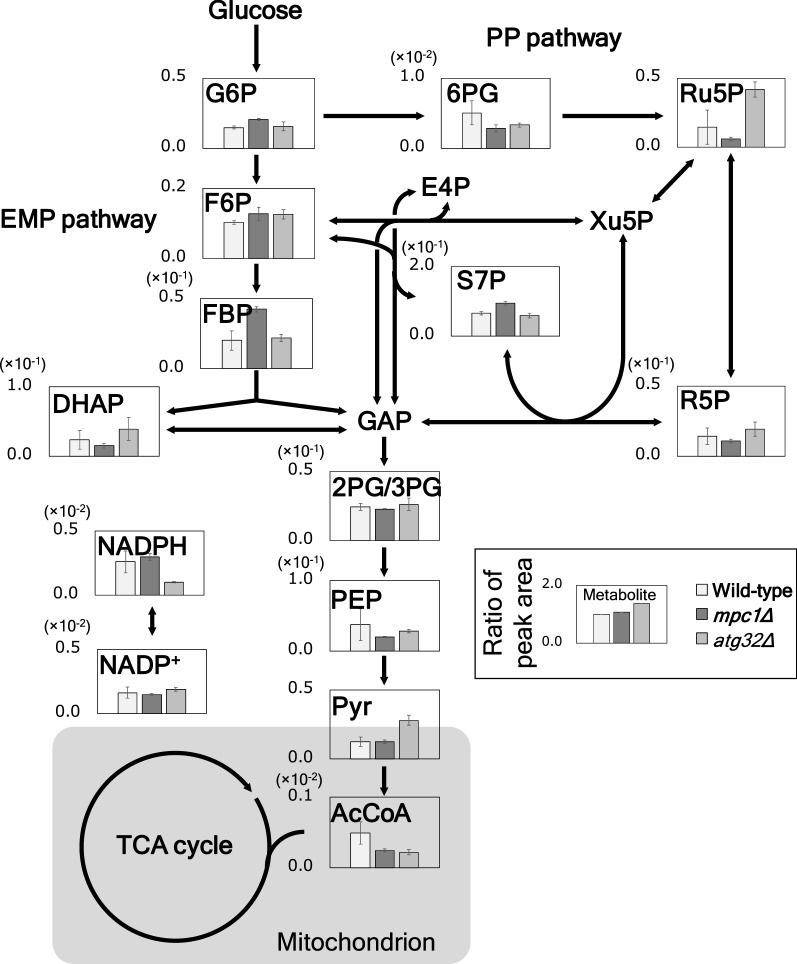
Metabolome analysis of glycolysis in SCM001 (wild-type), SCM053 (*mpc1Δ*), and SCM003 (*atg32Δ*). The vertical axis presents the relative peak area of each metabolite to that of the internal standard (dCS). Error bars represent the standard deviation from three replicate fermentations

Figure 3 presents the relative quantitation of the pool size of glycolytic metabolites for the wild-type (SCM001), *mpc1Δ*, and *atg32Δ* strains (SCM053 and SCM003). On comparing both knockout strains, with similar changes in the metabolic flux distributions, the knockout of *MPC1* and *ATG32* caused distinct effects on the intracellular pool size. Knockout of *MPC1* indicated a 2.1-fold increase in fructose 1,6-bisphosphate (FBP) and 50% decrease in phosphoenol pyruvate (PEP) compared to the wild-type strain. Since FBP is an allosteric activator of pyruvate kinase (PK) that converts PEP to pyruvate [20], the increased FBP concentration led to high PK activity, resulting in reduced PEP concentration. This was in accordance with the growth increase and ethanol production. Moreover, the acetyl-CoA pool size was reduced in *mpc1Δ* strain suggesting that the knockout of *MPC1* inhibited the mitochondrial pyruvate uptake and metabolism.

In contrast, intracellular acetyl-CoA pool size was also decreased, the pyruvate accumulation was observed in *atg32Δ* strain (2.3-fold higher than that of the control strain). Moreover, the NADPH pool size decreased by 0.4-fold. Because the NADPH was consumed as a response to oxidative stress, the degree of ROS activities was measured using a fluorescent reagent (Additional file 1: Figure S2). No change was observed in the fluorescence intensity of the control and *mpc1Δ* strains (733 ± 35 and 752 ± 36, respectively). Nevertheless, as the fluorescence intensity increased in *atg32Δ* strain (929 ± 36), the increase in ROS activity was confirmed. Therefore, in the *atg32Δ* strain, NADPH was consumed by response to oxidative stress, despite the increase of high PP pathway flux. On the other hand, no oxidative stress was observed in the *mpc1Δ* strain, in which the NADPH pool was increased. Thus, although both mutant strains displayed similar flux distribution in their central metabolism, the differences in the responses to oxidative stress and fluxes that were not included in the central metabolism were likely to affect the metabolite pool sizes. Furthermore, in the *atg32Δ* strain, mitochondrial metabolic activities decreased because of the oxidative stress due to the inhibition of mitophagy for the degradation of malfunctioning mitochondria. These findings were consistent with the decrease in TCA cycle flux and pyruvate accumulation.

### Application of intracellular excess pyruvate to 2,3-butanediol production

Both gene knockout of *MPC1* and *ATG32* were used for repressing the mitochondrial metabolism. To investigate the effect of these gene knockouts on the cytosolic production of pyruvate-derived chemicals, 23BD producing yeast strains were constructed. The 23BD is biologically synthesized from two molecules of pyruvate by four-step reactions catalyzed by acetolactate synthase (ALS), acetolactate decarboxylase (ALDC), and butanediol dehydrogenase (BDH). In this study, a plasmid constructed in the previous study (pATP426-als^Lp^_Op_-aldc^Ll^_Op_-BDH1, *als*^*Lp*^_*Op*_, *acdc*^*Ll*^_*Op*_, *BDH1*: codon-optimized ALS and ALDC genes derived from *Lactobacillus plantarum* and *Lactococcus lactis* and the BDH genes derived from *S. cerevisiae* [6]) was introduced in *S. cerevisiae* BY4742 (SCM043 strain) for cytosolic 23BD production.

**Fig. 4:**
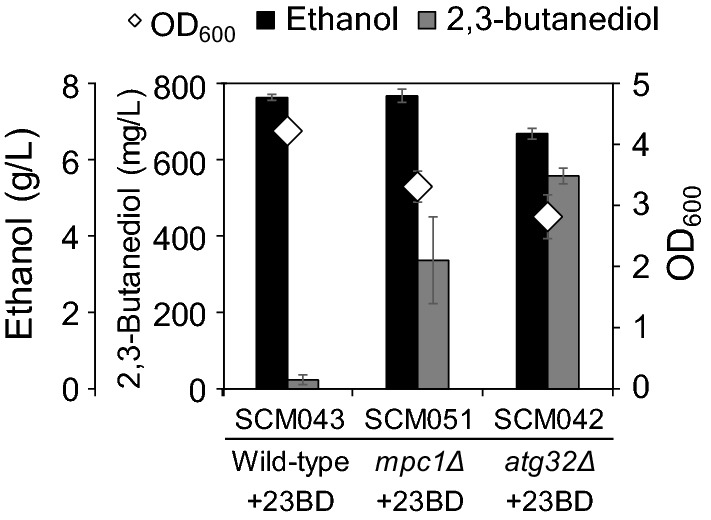
Test-tube scale culture of SCM043 (wild-type), SCM051 (*mpc1*Δ), SCM042 (*atg32*Δ) strains for 2,3-butanediol fermentation. The black and gray bars represent ethanol and 2,3-butanediol titers, respectively. +23BD denotes the strains harboring the plasmid pATP426-als^Lp^_Op_-aldc^Ll^_Op_-BDH1 for 23BD production. Open diamonds represent the OD_600_ of strains. Each value represents the standard deviation of three replicate fermentations

The strains were cultivated in 5 mL of SD medium in test tubes with initial OD_600_ value as 1 (Fig. 4). The 23BD titer of SCM043 strain harboring a plasmid pATP426-als^Lp^_Op_-aldc^Ll^_Op_-BDH1 was 23.5 ± 12.8 mg/L. The additional knockout of *MPC1* and *ATG32* (SCM051 and SCM042) increased the 23BD production strain titer by 14.3-fold and 23.6-fold (336.4 ± 113.5 mg/L and 557.0 ± 20.6 mg/L, respectively). In contrast, the final OD_600_ values decreased to 3.3 ± 0.3 and 2.6 ± 0.4 when compared to the SCM043 strain (4.2 ± 0.1). These results indicated that the *ATG32* knockout was more effective than the *MPC1* knockout for 23BD production, although both gene knockouts could redirect the saved carbon flux of mitochondria during 23BD production.

### Growth-arrested high-density fermentation for 23BD production

**Fig. 5:**
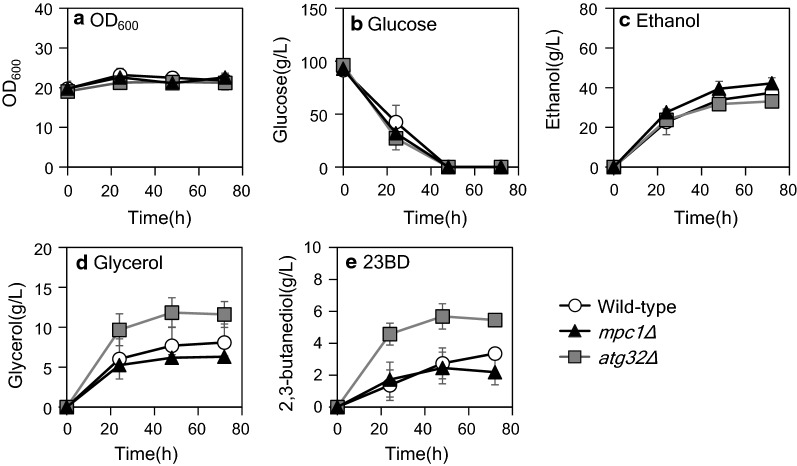
High-density fermentation test of 2,3-butanediol production strains. **a** OD_600_ and **b** glucose, **c** ethanol, **d** glycerol, and **e** 2,3-butanediol concentrations of strain SCM043 (wild-type, Open circle), SCM051 (*mpc1Δ*, closed triangle), and SCM042 (*atg32Δ*, gray square). Error bars represent the standard deviations from three replicate fermentations

Anaerobic high-density fermentation was carried out in 50 mL culture of 100 g/L glucose SD medium with an initial OD_600_ = 20 (Fig. 5). Cell growth was arrested in this condition since the OD_600_ value was not significantly changed during the 72 h of fermentation. The entire glucose was consumed at 48 h; the 23BD titer of the control strain (SCM043 strain) at 72 h culture was 3352.6 ± 263.9 mg/L. SCM042 strain improved 23BD titer to 5452.6 ± 359.8 mg/L by *ATG32* knockout; however, knockout of *MPC1* decreased the titer to 2184.4 ± 796.8 mg/L (SCM051 strain). In contrast, *mpc1Δ* strain indicated 42.2 ± 2.8 g/L of ethanol production titer compared to the control strain (37.4 ± 4.4 g/L) and the *atg32Δ* strain (33.1 ± 1.3 g/L).

## Discussion

Herzig et al. and Bricker et al. [21, 22] reported that the growth of *mpc1Δ* strain was improved by the plate cultures with supplementation of leucine and valine. However, in this study, the *mpc1Δ* and *atg32Δ* strains grew better compared to the wild type strain in test tube culture when supplemented only with leucine. Knockout of *MPC1* was confirmed by PCR (Additional file 1: Figure S3). Therefore, we conducted a drop test to compare the discrepancy of growth abilities between our mutants and mutants of prior studies (Additional file 1: Figure S4). The growth of the *MPC1* mutant was improved by the addition of 100 mg/L valine and 100 mg/L leucine compared to the wild type strain. The *MPC1* mutant supplemented with valine and leucine behaved similarly as previously described. However, the growth rate of the control strains was markedly different, probably due to the difference of strains and unexpected mutations. Therefore, we conclude that the growth of our *mpc1Δ* strain was not different from previous studies’ growth.

In the present study, ^13^C-MFA was carried out to investigate the metabolic perturbations in *mpc1Δ* and *atg32Δ* strains. The carbon fluxes of the TCA cycle were successfully suppressed (Fig. 2) and redirected for cell growth (Table 2). Although the reduction in the TCA cycle flux and the increased growth rate contradicted each other, yeast metabolisms were considered as anaerobic reactions due to the Crabtree effect as the strains were cultured in 20 g/L of glucose SD medium. Thus, in the control strain, TCA cycle presumably worked with alternative respiration, which produced water by transferring electrons from NADH directly to oxygen without the electron transport chain and ATP generation. The alternative respiration also decreased the ethanol fermentation ability [23]. In these strains, the glycolysis acted as an ATP producer for growth improvement, and the decrease in TCA cycle flux increased the carbon quantity necessary for biomass formation.

The knockout of *MPC1* increased cytosolic 23BD production in test-tube scale fermentation coupled with cell growth (Fig. 4). Enhanced *MPC1* and *MPC2* expression increased the mitochondrial isobutanol titer in the previous study [12]. In the present study, MPC knockout indicated that it was effective in improving the pyruvate-derived chemical production in the cytosolic biosynthesis pathway. This result suggested that both the expression and knockout of MPC genes are essential for yeast metabolic engineering in certain situations; however, the ethanol titer was increased in high-density fermentation compared to the 23BD titer (Fig. 5e). These results suggested that there were competitions of the pyruvate utilization for biomass synthesis, ethanol, and 23BD production. In the *mpc1Δ* strain, the Crabtree effect may have strongly enhanced and activated ethanol production under the high-density anaerobic condition. Therefore, the expression of powerful enzymes for 23BD production or decreased activity of the ethanol biosynthesis pathway was required for the improvement of 23BD titer in anaerobic conditions.

Shiroma et al. [15] reported that improvement in the ethanol titer in *Sake* brewing *ATG32* knockout strain occurred due to decreased biomass synthesis owing to lack of the reuse of nutrient sources available after the mitochondrial degradation. Nevertheless, in the present study, the relation between the growth rate and the ethanol production was not consistent, indicating the contribution of reduced mitochondrial flux for the increase of growth abilities (Table 2, Fig. 2). 23BD fermentation test also indicates that the distribution of the pyruvate consumption flux in *atg32Δ* strain was controlled by the delicate balance between the pathways and influenced sensitively by tugging power of metabolic enzymes like *mpc1Δ* strain. However, the influence of the Crabtree effect in the *atg32Δ* strain was reduced under anaerobic conditions of high-density culture compared to the *mpc1Δ* strain. This might be due to the preservation of the pyruvate transportability in the *atg32Δ* strain, wherein ethanol production was suppressed by trapping of pyruvate in mitochondria, followed by the conversion of pyruvate to acetolactate by the endogenous ALS activity encoded by *ILV2*, an intermediate of 23 BD.

## Conclusion

In the present study, strategies for the repression of mitochondrial metabolism, knockout of the mitochondrial pyruvate carrier gene (*MPC1*), and the essential gene for mitophagy (*ATG32*) were assessed in order to improve the pyruvate-derived chemical productivity in yeast. Both gene knockouts successfully suppressed the carbon flux of TCA cycle present in the mitochondria and redirected the carbon flux to the cell growth. The analysis of the intracellular metabolites suggested that the knockout of *MPC1* inhibited mitochondrial pyruvate uptake and metabolism, whereas that of *ATG32* decreased mitochondrial metabolism by oxidative stress due to the mitophagy inhibition. These findings were applied in the cytosolic 23BD production and it was confirmed that both gene knockouts were effective in improving the production titers. The results of this study suggested that the new approaches in metabolic engineering involving the mitochondrial transporters and membrane dynamics are effective in controlling the metabolism to improve the cytosolic target chemical productivity.

## Methods

### Strains, plasmids, and yeast transformation

The yeast strains and plasmids used in this study are listed in Table 1. *S. cerevisiae* BY4742 (*MATα*, *his3Δ1*, *leu2Δ0*, *lys2Δ0*, *ura3Δ0*; purchased from Thermo Scientific, Pittsburgh, PA, USA) was used as the yeast host strain. Plasmids were derived from the pGK and pATP vectors, in which the gene expression is controlled by the *PGK1*, *ADH1*, and *TDH3* promoters [21, 22].

### Culture conditions

All strains were cultured in yeast extract peptone dextrose (YPD) medium (10 g/L bacto yeast extract, 20 g/L bacto peptone, 20 g/L glucose) and synthetic dextrose (SD) medium (67 g/L yeast nitrogen base without amino acids and 20 g/L glucose, as necessary, 60 mg/L leucine, 30 mg/L lysine hydrochloride, 20 mg/L histidine, and 20 mg/L uracil). In the carbon labeling experiments, SD medium (67 g/L yeast nitrogen base without amino acids and 20 g/L [1-^13^C] glucose) containing the required amino acids was used. [1-^13^C] glucose (99%) was purchased from Cambridge Isotope Laboratories (Andover, MA, USA). Yeast cells from the plate medium were cultured in 5 mL of SD medium containing the required amino acids at 30 °C by shaking at 150 rpm. In the main culture, cells were incubated with similar conditions. The initial OD_600_ values were set at 0.5. The OD_600_ values were determined via a spectrophotometer (UVmini-1240, Shimadzu, Kyoto, Japan). For the fermentation test, transformants were cultured for 72 h at 30 °C by shaking at 150 rpm in 5 mL of SD medium containing 20 g/L glucose and required amino acids. The initial OD_600_ values for the main cultures were set at 1.0.

For high-density fermentation, cells were inoculated into 50 mL of synthetic medium (67 g/L yeast nitrogen base without amino acids and 100 g/L glucose, as necessary, 60 mg/L leucine, 30 mg/L lysine hydrochloride, 20 mg/L histidine, and 20 mg/L uracil). The initial OD_600_ values were set at 20. Fermentation was carried out at 30 °C with mild agitation in 100-mL closed bottles equipped with a bubbling CO_2_ outlet.

### Analysis of extracellular metabolites

To determine the concentrations of glucose, ethanol, acetate, glycerol, and pyruvate in the culture medium, supernatant was obtained by centrifugation at 15,000 rpm at 4 °C for 5 min and was subjected to gas chromatography (GC; Agilent 7890A GC; Agilent Technologies, Santa Clara, USA) and high-performance liquid chromatography (HPLC) system (Shimadzu, Japan). The GC was operated with the following conditions: column, Stabilwax 60 m × 0.32 mm ID × 1 μm (Restek, Bellefonte, USA); carrier gas, helium; flow rate, 6.5 mL/min; injection volume, 1 μL; split ratio, 1:10; oven temperature, 70 °C for 3 min and raised at 10 °C/min; flame ionization detector (FID) temperature, 250 °C. The HPLC system was equipped with an Aminex HPX-87H column (7.8 mm, 300 mm, Bio-Rad, USA), UV/vis detector (SPD-20A, Shimadzu, Kyoto, Japan), and refractive index detector (RID-10A, Shimadzu, Kyoto, Japan). The column temperature was set at 65 °C, and 1.5 mM H_2_SO_4_ was used as the mobile phase with a flow rate of 0.5 mL min^−1^. The flow cell temperature of the refractive index detector was set at 40 °C.

### ^13^C-metabolic flux analysis (^13^C-MFA)

To analyze the ^13^C label patterns of amino acids derived from cellular proteins, the cells in the exponential growth phase were collected via centrifugation at 15,000 rpm at 4 °C for 5 min. The cell pellet was washed twice with 0.9% NaCl and was hydrolyzed in 2 mL 6 N HCl at 105 °C for 18 h. After filtration (Cosmonice Filter W; pore size, 0.45 µm; filter diameter, 13 mm; Nacalai Tesque, Kyoto, Japan), 10 µL of the internal standard (600 µM cycloleucine) was added to the hydrolysate and evaporated to dryness. The dried residue was dissolved in 50 µL acetonitrile and 50 µL *N*-(tert-butyldimethylsilyl)-*N*-methyl-trifluoroacetamide containing 1% tert-butyldimethylchlorosilane and incubated at 105 °C for 1 h. After cooling overnight, the supernatant was subjected to gas chromatography-mass spectrometry (GC–MS) analysis following a previously described method [24]. The metabolic network of *S. cerevisiae* used the previously published models [25]. The metabolic model includes the major pathways of central carbon metabolism (glycolysis, PP pathway, anaplerosis, and tricarboxylic acid cycle) and the transport reaction between cytosol and mitochondria. Pyruvate, oxaloacetate, and acetyl-CoA (AcCOA) in the cytosol and mitochondria are considered as separate metabolite pools.

The computational procedure was performed by OpenMebius software [26] using MATLAB2013a (MathWorks, Natick, MA, USA). The metabolic flux distribution was estimated by nonlinear fitting of the metabolic model using the isotopic labeling patterns of proteinogenic amino acids determined by GC–MS. The nonlinear optimization was performed by using “fmincon” function in MATLAB optimization toolbox (MathWorks, Natick, MA, USA).

A 95% confidence interval of citrate synthase (CIT) flux was calculated by a grid search method following a previously described method [25, 27–29].

### Liquid chromatography-tandem–mass spectrometry (LC–MS/MS) analysis of intermediate metabolites

Cells in the exponential growth phase were inoculated into a fresh medium with initial OD_600_ = 1 and were harvested after 24 h for the LC–MS/MS analysis of extracted glycolytic intracellular metabolite pools ([OD_600_ value] × [volume (mL)] = 20). The culture broth was sampled rapidly and filtered through a filter of 0.5 μm pore size (PTFE-type membrane, Advantec, Tokyo, Japan). Cells on the filter were immediately immersed in 1.6 mL methanol with 10 mM of 10-camphor sulfonic acid (dCS; − 80 °C) and were preserved at − 80 °C until further extraction [30, 31]. Metabolites were extracted by adding 1.6 mL of chloroform (− 30 °C) and 640 μL of Milli-Q water (4 °C) and vortexed for 1 min; thereafter, the mixture was centrifuged at 4500×*g* for 40 min at 4 °C. Two hundred and fifty microliters of aqueous layer were dispensed into a 1.5-mL tube. The extract solution was dispensed into five Eppendorf tubes and dried using a SpeedVac SPD1010 (Thermo Fisher Scientific, Waltham, MA, USA) at room temperature. The dried samples were suspended in 50 μL of Milli-Q water.

LC–MS/MS analysis (LC: Agilent 1200 infinity series; Agilent Technologies, Santa Clara, CA, USA; MS/MS: API 3200; AB SCIEX, MA, USA) was performed under the following conditions [32]: column, ProteCol-P C18 HQ103 (2.1 × 150 mm, particle size of 3 μm); mobile phase, 10 mM tributylamine/15 mM acetic acid in water (A) and methanol (B); flow rate: 0.2 mL min^−1^; gradient curve, 100% A: 0% B at 0 min, 10% A: 90% B at 24 min, 100% A: 0% B at 24.1 min, and 100% A: 0% B at 30 min; injection volume, 3 μL; column temperature, 35 °C; mode of mass analysis, negative ion mode; nebulizer flow, 55 psi; dry gas flow rate, 10 L min^−1^; sheath gas flow rate, 11 L min^−1^; dry gas temperature, 300 °C; sheath gas temperature, 380 °C; capillary voltage, 3.5 kV. The parameters for selected reaction monitoring (SRM) of target metabolites have been specified in Additional file 1: Table S2. The peak of each target metabolite was identified by comparing its chromatographic behavior with that of an authentic standard. The peak area was determined using Analyst software (version 1.6.2, AB SCIEX). Relative quantitation was performed based on the MS data using the ratio of each metabolite’s peak areas to the dCS peak area.

### Measurement of intracellular oxidative stress of the yeast cell

Intracellular oxidative stress was measured using an OxiSelect™ Intracellular ROS assay kit (Cell Biolabs Inc.). Cells were harvested (OD_600_ × mL = 1) and centrifuged. The cell pellet was washed by 1 mL of PBS buffer twice and incubated in 1 mL of SD medium containing 40 µL of 2,7-dichlorodihydrofluorescin diacetate (DCFH-DA), 30 °C for 60 min. Cells were harvested and washed by PBS again and were then suspended in 100 µL of PBS on the 96 well plate for measuring oxidized DCF green fluorescent.

## Supplementary information

**Additional file 1: Additional tables and figures.**

## Data Availability

The datasets used in the current study are available from the corresponding author on reasonable request.

## Abbreviations

G6P: glucose 6-phosphate
F6P: fructose-6-phosphate
FBP: fructose-1,6-bisphosphate
DHAP: dihydroxyacetone phosphate
GAP: glyceraldehyde 3-phosphate
3PG: 3-phosphoglycerate
2PG: 2-phosphoglycerate,
PEP: phosphoenol pyruvate
Pyr: pyruvate
AcCoA: acetyl-CoA
Ru5P: ribulose-5-phosphate
Xu5P: xylose-5-phosphate
R5P: ribose 5-phosphate
E4P: erythrose-4-phosphate
S7P: sedoheptulose-7-phosphate
αKG: α-ketoglutarate
Mal: malate
Oxa: oxaloacetate
CIT: citrate synthase
EMP pathway: Embden–Meyerhof–Parnas pathway
PP pathway: pentose-phosphate pathway
MPC: mitochondrial pyruvate carrier
TCA cycle: tricarboxylic acid cycle
EtOH: ethanol
Glyc: glycerol
23BD: 2,3-butanediol
