# Supplementary material for: Repression of mitochondrial metabolism for cytosolic pyruvate-derived chemical production in Saccharomyces cerevisiae
Source: Microb Cell Fact. 2019 Oct 15;18:177. doi: 10.1186/s12934-019-1226-6 (PMC6794801; doi:10.1186/s12934-019-1226-6)
Supplement: Supplementary file 1 — Additional file 1: Additional tables and figures. [file 12934_2019_1226_MOESM1_ESM.docx]

# **Additional file 1**

# **Tables**

## Table S1 Metabolic model for ^13^C-metabolic flux analysis

| Reaction | Reaction | Carbon transition |
| --- | --- | --- |
| HEX1 | Subs_Glc --> G6P | ABCDEF --> ABCDEF |
| r2 | G6P --> F6P | ABCDEF --> ABCDEF |
| r3 | F6P --> G6P | ABCDEF --> ABCDEF |
| r4 | F6P --> DHAP + GAP | ABCDEF --> CBA + DEF |
| r5 | DHAP --> GAP | ABC --> ABC |
| r6 | GAP --> DHAP | ABC --> ABC |
| r7 | GAP --> 3PG | ABC --> ABC |
| r8 | 3PG --> GAP | ABC --> ABC |
| r10 | Ser --> Gly + CO2_in | ABC --> AB + C |
| r11 | 3PG --> PEP | ABC --> ABC |
| r12 | PEP --> 3PG | ABC --> ABC |
| r13 | PEP --> PYRcyt | ABC --> ABC |
| r14 | PYRcyt --> PEP | ABC --> ABC |
| r15 | PYRcyt + CO2_in --> OAAcyt | ABC + D --> ABCD |
| r17 | OAAcyt --> Thr | ABCD --> ABCD |
| r18 | Thr --> Gly + AcAl | ABCD --> AB + CD |
| r19 | PYRcyt --> AcAl + CO2_in | ABC --> BC + A |
| r20 | PYRmit --> AcCOAmit + CO2_in | ABC --> BC + A |
| r21 | AcCOAmit + OAAmit --> IsoCitmit | AB + CDEF --> FEDBAC |
| r22 | IsoCitmit --> aKGmit + CO2_in | ABCDEF --> ABCDE + F |
| r23 | aKGmit --> Sym_Sucmit | ABCDE --> BCDE + A |
| r24 | Sym_Sucmit --> Malmit | ABCD --> ABCD |
| r25 | Malmit --> Sym_Sucmit | ABCD --> ABCD |
| r26 | Malmit --> OAAmit | ABCD --> ABCD |
| r27 | OAAmit --> Malmit | ABCD --> ABCD |
| r28 | Malmit --> PYRmit + CO2_in | ABCD --> ABC + D |
| r29 | OAAcyt --> OAAmit | ABCD --> ABCD |
| r30 | OAAmit --> OAAcyt | ABCD --> ABCD |
| r31 | AcAl --> Acetate | AB --> AB |
| r32 | Acetate --> AcCOAcyt | AB --> AB |
| r33 | Ru5P --> R5P | ABCDE --> ABCDE |
| r34 | R5P --> Ru5P | ABCDE --> ABCDE |
| r35 | Ru5P --> Xu5P | ABCDE --> ABCDE |
| r36 | Xu5P --> Ru5P | ABCDE --> ABCDE |
| r37 | E4P + Xu5P --> F6P + GAP | ABCD + EFGHI --> EFABCD + GHI |
| r38 | GAP + F6P --> Xu5P + E4P | GHI + EFABCD --> EFGHI + ABCD |
| r39 | GAP + S7P --> F6P + E4P | ABC + DEFGHIJ --> DEFABC + GHIJ |
| r40 | E4P + F6P --> S7P + GAP | GHIJ + DEFABC --> DEFGHIJ + ABC |
| r41 | R5P + Xu5P --> S7P + GAP | ABCDE + FGHIJ --> FGABCDE + HIJ |
| r42 | GAP + S7P --> Xu5P + R5P | HIJ + FGABCDE --> FGHIJ + ABCDE |
| r43 | AcCOAcyt --> AcCOAmit | AB --> AB |
| r44 | AcCOAmit --> AcCOAcyt | AB --> AB |
| r45 | PYRmit --> Ala | ABC --> ABC |
| r46 | PYRcyt --> Ala | ABC --> ABC |
| G6P_B | G6P --> [Biomass] |  |
| F6P_B | F6P --> [Biomass] |  |
| R5P_B | R5P --> [Biomass] |  |
| E4P_B | E4P --> [Biomass] |  |
| Glycerol_ex | DHAP --> [Glycerol] |  |
| DHAP_B | DHAP --> [Biomass] |  |
| GAP_B | GAP --> [Biomass] |  |
| Ser_B | Ser --> [Biomass] |  |
| Gly_B | Gly --> [Biomass] |  |
| PEP_B | PEP --> [Biomass] |  |
| Thr_B | Thr --> [Biomass] |  |
| OAAcyt_B | OAAcyt --> [Biomass] |  |
| EtOH_ex | AcAl --> [EtOH] |  |
| Acetate_ex | Acetate --> [Acetate] |  |
| AcCOAcyt_B | AcCOAcyt --> [Biomass] |  |
| AcCOAmit_B | AcCOAmit --> [Biomass] |  |
| PYRmit_B | PYRmit --> [Biomass] |  |
| Ala_B | Ala --> [Biomass] |  |
| aKGmit_B | aKGmit --> [Biomass] |  |
| CO2_uptake | Subs_CO2 --> CO2_in | A --> A |
| CO2_ex | CO2_in --> [CO2_ex] |  |
| r1 | G6P --> Ru5P + CO2_in | ABCDEF --> BCDEF + A |
| r9 | 3PG --> Ser | ABC --> ABC |
| r16 | PYRcyt --> PYRmit | ABC --> ABC |

## Table S2 Optimized conditions for analytical validation of ion-pairing liquid chromatography triple-stage quadruple mass spectrometry for determining the target compounds

| Metabolites | Selected reaction monitoring transitions | Retention time(min) | Declustering potential(V) | Collision energy(V) | Collision cell exit potential(V) |
| --- | --- | --- | --- | --- | --- |
| G6P | 259/97 | 12.81 | -30 | -22 | -4 |
| F6P | 259/97 | 12.81 | -30 | -22 | -4 |
| FBP | 339/97 | 20.94 | -35 | -76 | -4 |
| DHAP | 169/97 | 16.06 | -20 | -16 | -4 |
| 6PG | 275/79 | 20.64 | -35 | -72 | -4 |
| Ru5P | 229/97 | 13.32 | -20 | -22 | -4 |
| R5P | 229/97 | 13.32 | -20 | -22 | -4 |
| S7P | 289/97 | 13.29 | -35 | -30 | -4 |
| 3PG+2PG | 185/79 | 20.72 | -25 | -20 | -4 |
| PEP | 167/79 | 21.17 | -25 | -22 | -4 |
| PYR | 87/43 | 15.95 | -25 | -14 | -4 |
| AcCoA | 808.3/79 | 23.7 | -125 | -122 | -4 |
| NADPH | 744.3/79 | 22.69 | -153 | -130 | -4 |
| NADP+ | 742.2/620.1 | 30 | -25 | -24 | -16 |
| dCS | 230.979/79.9 | 23 | -60 | -64 | -4 |

# **Figures**

## **Figure S1 Fermentation profiles of BY4742 wild-type, *atg32Δ*, and *mpc1Δ* strains.** Fermentation profiles are depicted for (a) SCM001 (wild-type), (b) SCM053 (*mpc1Δ*), and (c) SCM003 (*atg32Δ*) strains in test-tube culture. (Open circle: OD_600_, closed circle: glucose concentration, gray diamond: glycerol concentration, and gray triangle: ethanol concentration). Error bars represent the standard deviations from three replicate fermentations.

## **Figure S2 Measurement of intracellular oxidative stress in BY4742 wild-type, *atg32Δ*, and *mpc1Δ* strains.** Intracellular oxidative stress in SCM001 (wild-type), SCM053 (*mpc1Δ*), and SCM003 (*atg32Δ*) strains were measured using 2,7-Dichlorodihydrofluorescin diacetate (DCFH-DA). Error bars represent the standard deviations from three replicate colonies.

## **Figure S3 PCR experiment to confirm *MPC1* knockout in BY4742 wild-type (SCM001, WT) and *mpc1Δ* strains (SCM053, *mpc1Δ*).** MW denotes molecular weight. For PCR amplification, the following primers were used: MPC1_A: CATGGCTAGTCAAATAAGTGGAGTT, MPC1_D: AATACAAAGGAGATGAGAGGGAAAT. In the SCM053 strain, *MPC1* (935 bp) was replaced by the *KanMX* cassette (2126 bp).

## **Figure S4 Comparison of the growth of WT (SCM001), atg32Δ (SCM003), and mpc1Δ (SCM053) strains supplemented with valine and leucine**. For the drop test, the culture broth of each strain was harvested by an overnight test tube culture. Six microliters of the culture broth was dropped on the SD plate medium containing 20 mg/L histidine, 30 mg/L lysine hydrochloride, and equal concentrations of valine and leucine. The optical density was set to 0.5, 0.1, 0.02, 0.004, 0.0008, 0.00016, reading from the left. Cells were cultured for 2 days.
